# Considerations about the Bibliometric Impact Factor. The BJCVS is on the Right Track

**DOI:** 10.21470/1678-9741-2019-0602

**Published:** 2019

**Authors:** Paulo Roberto B. Evora, Andreia C. Feitosa do Carmo, Camila Sáfadi Alves Gonçalves, Domingo M. Braile

**Affiliations:** 1 Editor-in-Chief Interim - BJCVS Faculdade de Medicina de Ribeirão Preto da Universidade de São Paulo (FMRP-USP), Ribeirão Preto, SP, Brazil.; 2 Hospital São Paulo, Escola Paulista de Medicina da Universidade Federal de São Paulo, (EPM - UNIFESP), São Paulo, SP, Brazil.; 3 Sociedade Brasileira de Cirurgia Cardiovascular, São Paulo, SP, Brazil.; 4 Editor-in-Chief - BJCVS Faculdade de Medicina de São José do Rio Preto (FAMERP), São José do Rio Preto, SP, Brazil and Universidade de Campinas (UNICAMP), Campinas, SP, Brazil.

Bibliometric indicators are qualitative methods that allow the evaluation of scientific production through statistical techniques, and one of the most important indicators is the impact factor. The basis for the impact factor is the Journal Citation Report (JCR). Available from Clarivate Analytics, it was developed by Eugene Garfield in 1958, whose idea was to allow a practical analysis of the propagation of scientific information in their respective areas of knowledge. To do this, Garfield determined a numerator: the number of citations in the current year for any items published in the journal in the previous two years; and the denominator: the number of articles published in those two years^[1,2]^. The basic elements can be adapted to consider shorter or longer time intervals, according to the area of knowledge.

The metric was developed for responsible use in journal management, allowing librarians to evaluate and select which journals to sign and/or discard, and editors to track and check the evolution of their journals^[3,4]^. However, the impact factor is also being used to evaluate research performance and is applied at all organizational levels: authorship, institution, country/region, research field or journal, which distorts its application. To compensate for this, in 2018 the JCR reviewed journal profiles with a richer data context. One example is the bar chart that provides the value of the impact factor in the Percentile Rank in Category item, allowing the quartile to be viewed quickly.

When analyzing the impact factor data from Brazilian Journal of Cardiovascular Surgery (BJCVS), they were promising: the impact factor was 0.805 (Figure 1) and the journal is classified in the following categories: Cardiac & Cardiovascular System and Surgery in Q4 quartile (Figure 2). Regarding the contribution by country/region and organization, the highlight is for Brazil, which shows that we are still the majority of authors and readers of the journal (Figure 3).

**Fig. 1:**
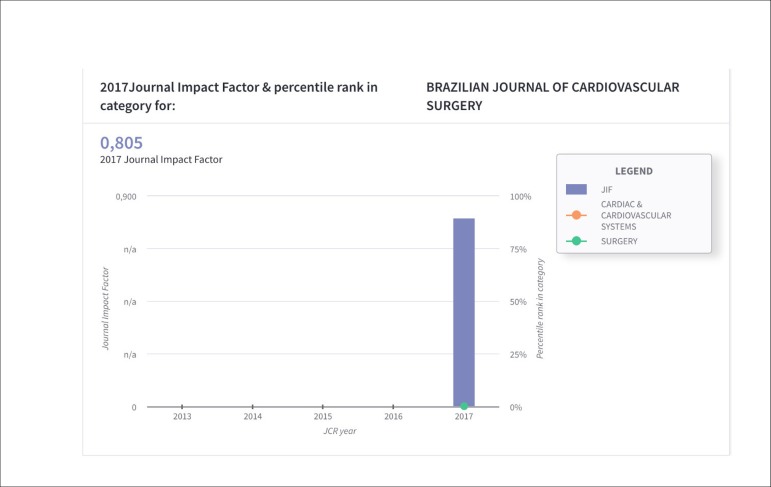
Impact Factor of BJCVS.

**Fig. 2:**
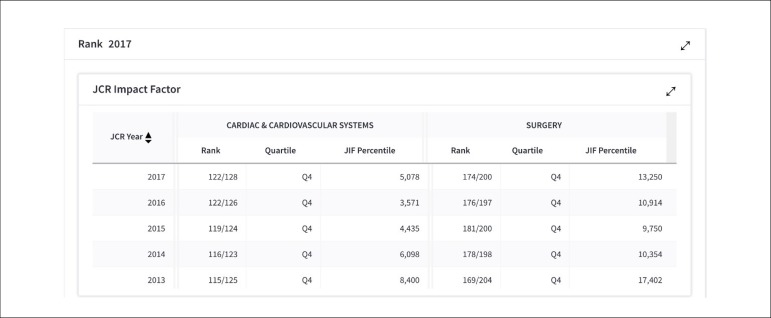
Category and Rank.

**Fig. 3:**
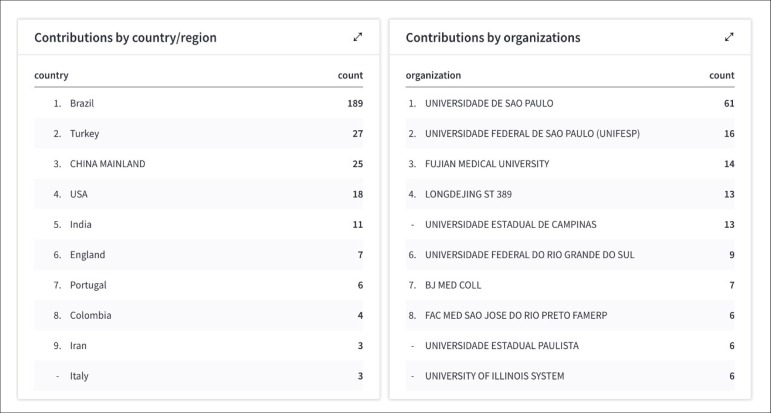
Contribution by region and organizations.

BJCVS believes it is on the right track by prioritizing the quality of its publications and a structure that protects the authenticity of published work, what is reflected in the journal indicators.

## Articles in this Issue

This issue of BJCVS presents a blind peer-reviewed selection of 20 papers that were selected by order of acceptance: 11 original papers, 1 review article, 4 selected case reports and 1 letter to the editor.

**Paulo Roberto B. Evora** https://orcid.org/0000-0001-9631-946X ^1^Editor-in-Chief Interim - BJCVS Faculdade de Medicina de
Ribeirão Preto da Universidade de São Paulo (FMRP-USP), Ribeirão
Preto, SP, Brazil. **Andreia C. Feitosa do Carmo** https://orcid.org/0000-0002-0387-7946 ^2^Hospital São Paulo, Escola Paulista de Medicina da Universidade
Federal de São Paulo, (EPM - UNIFESP), São Paulo, SP, Brazil. **Camila Sáfadi Alves Gonçalves** https://orcid.org/0000-0003-0611-7229 ^3^Sociedade Brasileira de Cirurgia Cardiovascular, São Paulo, SP, Brazil. **Domingo M. Braile** https://orcid.org/0000-0001-7704-2258 ^4^Editor-in-Chief - BJCVS Faculdade de Medicina de São
José do Rio Preto (FAMERP), São José do Rio Preto, SP, Brazil and
Universidade de Campinas (UNICAMP), Campinas, SP, Brazil.
